# A Hospital based study on sudden sensorineural Hearing Loss: It’s audiological characteristics and prevalence

**DOI:** 10.12669/pjms.37.4.3851

**Published:** 2021

**Authors:** Erum Naz, Ghulam Saqulain, Nazia Mumtaz, Muhammad Naveed Babur

**Affiliations:** 1 Ms. Erum Naz, MPhil (Hearing Sciences) Audiologist, Audiology Unit, Lahore General Hospital, Lahore, Pakistan; 2 Dr. Ghulam Saqulain, F.C.P.S (Otorhinolaryngology) Head of Department, Department of Otorhinolaryngology, Capital Hospital PGMI, Islamabad, Pakistan; 3 Dr. Nazia Mumtaz, PhD (Rehabilitation Sciences) Head of Department of Speech Language Pathology, Faculty of Rehab & Allied Health Sciences, Riphah International University, Lahore, Pakistan; 4 Dr. Muhammad Naveed Babur, PhD (Rehabilitation Sciences) Professor and Dean, Faculty of Allied Medical Sciences, Isra University, Islamabad, Pakistan

**Keywords:** Hearing loss, Sensorineural hearing, Sudden sensorineural hearing loss

## Abstract

**Objectives:**

To analyze the prevalence & characteristics of sudden sensorineural hearing loss.

**Methods:**

This cross sectional study including n=377 cases of sensorineural hearing impairment, using non-probability convenience sampling, who fulfilled the selection criteria was conducted from 1^st^ July 2019 to 31^st^ October 2019. Study was conducted at Audiology section of ENT department, Lahore General Hospital, Pakistan. Sample included both genders, aged 17-70 years. Cases suffering from inflammatory or obstructive conditions of the external or middle ear and those who could not undergo pure tone audiometry were excluded from the study. Following consent for inclusion in study, data was collected using basic demographic and medical history sheet followed by Audiometric evaluation. Statistical Software for Social Sciences Version 20.0 was used for data analysis.

**Results:**

The prevalence rate of sudden sensorineural hearing loss of 14(3.7%) being significantly more common in males 11(78%) than females 3(22%) (p=0.05) & age group 15-35 years (p=0.001). It is commonly of severe or profound degree with downward sloping audiogram (p<0.05), however it is not associated with vertigo (p=0.32), tinnitus (p=0.08) with no side predilection (p=0.27).

**Conclusion:**

We conclude that the prevalence of SSNHL is 3.7% being significantly more prevalent in males and those aged15-25 years. It is mostly characterized by severe to profound degree of hearing loss with downward sloping audiogram with no associated vertigo, tinnitus and side predilection.

## INTRODUCTION

Sudden sensorineural hearing loss (SSNHL) is considered a medical emergency^1^ and is defined as Sensorineural Hearing loss (SNHL) of > 30db on three contiguous pure tone audiometric frequencies within 72 hours.^2^ SSNHL is relatively common complaint and of idiopathic origin in 90%, other etiologies being infection, ototoxicity, noise, trauma, autoimmune disease, malignancy, vascular, neurologic, metabolic, and even non-organic etiology.^3^

The exact incidence of Sudden SNHL is not known. Estimate of incidence show a wide range of 11-77 per 100,000 people per year,^4^ with equal gender distribution and can occur at any age.^5^ SSNHL can present with varying degree of hearing loss and can accompany tinnitus and vertigo.^5^ Majority of cases are idiopathic with unknown etiology.^2^,^6^ Risk factors are uncertain and Herpes Simplex type 1 viral infection has been implicated.^7^

SSNHL is a subjective symptom perceived by the patient and hence to determine prevalence of hearing loss (HL), its type and threshold of hearing, a pure-tone audiogram is required.^3^ Though majority (65%) cases of idiopathic SSNHL recover spontaneously,^8^ however cases need to be investigated for infective causes and to exclude lesions in cerebellopontine angle, internal auditory canal and inner ear.^8^ Early recognition and intervention can help recovery in thresholds of hearing,^6^ which depends on severity of HL, age, presence of vertigo and configuration of audiogram.^2^ Institution of early treatment with standard doses of oral steroids in cases with idiopathic SSNHL is recommended^8^ and may increase changes of recovery.

Acknowledging the available literature, the exact prevalence of SSNHL is not known with highly variable incidence reported in studies,^4^ with no local literature on its prevalence and characteristics. Hence the current study was conducted to explore the prevalence & characteristics of sudden sensorineural hearing loss. This study has significant importance as its finding may be helpful for researchers to conduct in depth research in the field with different objectives. SSNHL is topic of great importance and needs more research to be conducted in Pakistan.

## METHODS

This is a cross sectional study which recruited n= 377 patients with SNHL, who attended Audiology section of ENT Department of Lahore General Hospital, Pakistan using non probability convenience sampling technique over a period of four months extending from 1st July 2019 to 31st October 2019. Sample included both genders, aged 17-70 years. Cases with inflammatory and obstructive pathologies of external and middle ear, those who could not undergo audiological testing or were found to have conductive element were excluded. Study was conducted after obtaining approval of IRB of Isra Institute of Rehabilitation Sciences, Isra University vide Registration No 1609-M.Phil HS-007 dated 24^th^ June, 2019 and informed consent of the sample. A basic demographic sheet, medical history sheet and PTA were used to collect data.

History was noted in a confidential setting including duration of HL, associated complications like tinnitus, vertigo, exposure to ototoxic agents, occupational or leisure noise and treatment history. This was followed by physical examination including otoscopy to rule out any obstructive and inflammatory pathology and pure tone audiometry was done in cases suspected of having sensory neural hearing loss. Computer based Pure Tone Audiometer Labat, made in Italy was used and testing performed in sound proof room which met American National Standards Institute (ANSI) S 3.1-1991 standards. Both ears will be tested for air and bone conduction using the method of ascending pure tones at frequencies of 0.5, 1, 2, 4, 6 and 8 kHz before descending to 1 and 0.5 kHz. Bone conduction was checked at frequencies of 0.5 to 4 kHz

Data collected was analyzed using SPSS - Version 20.0. Descriptive statistics was used. Variables specially studied included degree and type of HL, ears affected, associated symptoms and age and gender. Pearson’s Chi-square test was used to determine association of type of HL with demographic and clinical variables. Independent sample t-test was used to see any significant difference in mean thresholds between the two ears. P-value of 0.05 was taken as significant.

## RESULTS

Sample of 377 cases, revealed 203 (53.8%) males and 174(46.2%) females with a ratio of 1.67:1 and mean age of 42.04±1.73 years (Table-I). Of the sample population of 377 cases, the prevalence of SSNHL was 14(3.71%) while the remaining 363 (96.29%) had chronic HL.

**Table-I:** Demographic & Clinical Variables* Type of Hearing Loss, Chi-Square Test. Cross Tabulation. (n = 377).

Characteristics of Variables		Type Of Hearing Loss		Total	Chi-Square

Variable	Characteristics	Acute (within 72 hours) (n=14)	chronic HL (n=363)	n(%)	X^2^ (P-value)
Age groups (Years)	15-35	12	133	145(38.5)	13.99 0.001
	36-55	2	145	147(39)	
	56-75	0	85	85(22.5)	
Gender	Male	11	192	203 (53.8)	3.57 0.05
	Female	3	171	174 (46.2)	
Vertigo	Present	0	24	24(6.4)	0.98 0.32
	Absent	14	339	353(93.6)	
Tinnitus	Present	8	125	133(35.3)	3.04 0.08
	Absent	6	238	244(64.7)	
Cause of Hearing Loss	Identified	10	70	80(21.2)	21.92 0.05
	Not Identified	4	293	297(78.8)	
Degree of Hearing loss in Right Ear	Normal hearing	1	50	51(13.5)	12.19 0.03
	Mild	0	48	48(12.7)	
	Moderate	0	68	68(18)	
	Moderately Severe	1	52	53(14.1)	
	Severe	4	47	519(13.5)	
	Profound	8	98	106(28.1)	
Degree of Hearing Loss in Left Ear	Normal hearing	5	46	51(13.5)	27.51 0.05
	Mild	2	68	70(18.6)	
	Moderate	0	73	73(19.4)	
	Moderately Severe	0	33	33(8.8)	
	Severe	6	32	38(10.1)	
	Profound	1	111	112(29.7)	
Ears Affected	Unilateral	5	84	89(23.6)	1.18 0.27
	Bilateral	9	279	288(76.4)	

Majority (n=12) cases of age group 15-35 years suffered SSNHL, while remaining (n=2) belonged to 36-55 years age group indicating significant (p=0.001) age association with SSNHL. Similarly gender association was noted (p=0.05) with 11 males and 3 females having SSNHL.

No significant association (p=0.32) of vertigo was noted with no case of vertigo among cases of SSHL. Similarly no significant association (P=0.081) of tinnitus was noted with eight out of 14 cases of SSNHL having tinnitus and 125 out of 363 cases of chronic hearing loss with tinnitus.

There was significant association (p=0.032) of severity of hearing loss in the right ear with maximum (n=8) cases having profound and n=4 having severe hearing loss among cases with SSNHL. On the other hand in the Left ear majority (n=6) had severe HL while n=5 had normal hearing in the group with SSNHL. While in the chronic HL cases majority (n=111) had profound HL. The difference was significant (p<0.001)

No significant difference (p-0.27) was noted as regards side affected in SSNHL and chronic HL groups. As regards threshold of hearing in cases with SSNHL study revealed a continuous rise from a Mean threshold of 54.28±28.22 at 250 Hz to 72.89±31.04 at 8 KHz in the Right Ear and from 54.88±28.62 at 250 Hz to 75.46+30.78 at 8 KHz in the Left Ear. However, t-test statistics did not reveal any significant difference at all the frequencies form 250- to 8 kHz (Table-I).

**Table-II:** Mean Thresholds of Hearing & T Test statistics for difference of mean thresholds between the two ears (n=14).

Ear	Frequency		Hearing Threshold		

	Hz	Minimum	Maximum	Mean	Std. Deviation
Right	250	15	105	54.28	28.22
	500	10	120	60.38	31.41
	1k	15	120	66.11	33.27
	2k	15	120	69.92	32.88
	4k	15	120	75.05	31.95
	8k	15	120	72.89	31.04
Left	250	15	120	54.88	28.62
	500	15	120	61.03	31.58
	1k	8	120	65.08	34.25
	2k	20	120	67.25	33.44
	4k	20	120	73.26	32.81
	8k	20	120	75.46	30.78

Frequency	Paired Differences (at 95% confidence interval)			t	p-value

Right Ear - Left Ear	Mean	Std. Deviation	Std. Error Mean		
	
250-250	-0.59	26.27	1.35	-0.43	0.66
500-500	-0.64	29.61	1.52	-0.42	0.67
1k-1k	1.03	30.90	1.59	0.64	0.51
2k-2k	2.66	29.88	1.53	1.73	0.08
4k-4k	1.79	29.85	1.53	1.16	0.24
8k-8k	0.96	27.98	1.61	0.59	0.55

## DISCUSSION

Sudden sensorineural hearing loss, recovers spontaneously in majority, hence exact incidence is not known with a wide range of 11 to 77 per 100,000 people affected per year.^4^ In the current study with a sample population of 377 cases, a low prevalence of SSNHL of 14(3.71%) was found. In contrast in an American study, a high incidence of 27 per 100000 annually have been reported by Alexander & Harris.^4^ The probable reason of low prevalence might be that majority of those affected delay consultation with a professional and hence recover and or don’t give much importance to HL, which is evident from the attitude of our population in which even cases of oral cancer present delay reporting for a medical advice ranging from one month to two years.^9^

In the current study of the 89 cases with unilateral HL, 5 had SSNHL, while of the 288 cases with bilateral HL 9 had SSNHL, however the difference was not statistically significant (p-0.27). In contrast to our study in which majority had bilateral HL, in a study by Atay G et al. in 45.9% cases right ear was affected and in 54.1% left ear was affected.^10^

Present research revealed that SSNHL is more prevalent in males as compared to females. With a total population of 203 (53.8%) males and 174(46.2%) females, 11 males and three females had SSNHL and difference was significant (p=0.05). Similarly, in study by Atay G et al. males were predominantly affected.^10^ While, Alexander & Harris^4^ noted a slight male predominance with ratio of 1.07:1 below 65 years and 1.30:1 above 65 years of age.

In the present study, SSNHL was significantly (p=0.001) more common in age group 15-35 years with 12 cases, while remaining two cases belonged to 36-55 years age group. In contrast Alexander and Harris, reported a higher incidence of 77/ 100000 cases at age 65 and above compared to 11/100000 below 18 years of age.^4^ Similarly higher prevalence in 43-53 years age group has been reported by Trevino Gonzalez JL et al,^5^ and by Lee HS et al. in 5th and 6th decade.^11^ The higher prevalence in younger age group in our study might be due to the fact that reduced hearing in elder adults in Pakistani community is considered a norm especially in the rural areas and are not commonly brought to hospital.

As regards etiology, different studies have reported widely varying prevalence of different etiological causes. According to Lloyd SKW et al. in 30% cases of SSNHL cause is identified including infection (44%), otologic disease (17%), neurologic and vascular (12%), malignancies (7%) and miscellaneous (4%), while the remaining 70% cases are idiopathic.^6^ In contrast majority (94.5%) of cases were idiopathic and in only 5.5% etiology was identified including acoustic neuroma, hemorrhage, inner ear malformations, and perilymph fistula in another study.^10^ In the current study, in majority (71.43%) cases cause was identified while in the remaining it was considered idiopathic. Also in majority the cause was trauma due to road traffic accident and fall from bike. In a meta-analysis by Chau et al. reported infections in 13%, aural causes in 5%, trauma in 4%, Vascular in 3%, tumors in 2% and other causes in another 2%.^12^ An interesting finding noted by Yen YC et al. was a threefold higher incidence in chronic otitis media cases.^13^ Also a higher incidence (1.3 fold) of SSNHL has also been reported in cases with cardiac rhythm disorders,^14^ Diabetes Mellitus,^15^ and hypo and hyperthyroidism.^16^ Here it must be kept in mind that identification of etiology is also related to the investigative facilities available and being used, which might result in difference in the frequency of different etiologies being reported from different centers.

In the present study there was significant association (p=0.032) of severity of hearing loss with type of HL with majority (eight cases) having profound SSNHL followed by (4 cases) with severe SSNHL in the right ear. On the other hand in the Left ear majority (6 cases) had severe SSNHL while (five cases) had normal hearing in the group with SSNHL. In this study the mean threshold of hearing in cases with SSNHL showed a continuous rise (Downward sloping audiogram) from a Mean threshold of 54.28±28.22 at 250 Hz to 72.89±31.04 at 8 KHz in the Right Ear and from 54.88±28.62 at 250 Hz to 75.46±30.78 at 8 KHz in the Left Ear. However, t-test statistics did not reveal any significant difference at all frequencies form 250- to 8kHz. In contrast in a study by Atay G et al. reported that majority (29.8%) had flat audiogram configuration, followed by downward sloping audiogram in 26% and upward sloping audiogram configuration in 22.7%. However profound degree of HL was noted in 21.5% cases of SSNHL.^10^

Common associated symptoms include aural fullness, vertigo and tinnitus.^6^ However, no significant association (p=0.32) of vertigo was noted with no case of vertigo among cases of SSNHL in current study. Also no significant association (p=0.081) of tinnitus was noted with 8 out of 14 cases of SSNHL having tinnitus and 125 out of 363 cases of chronic hearing loss having tinnitus. In contrast international literature indicates association of symptoms of tinnitus as well as vertigo with a high frequency.^2^ While vertigo did not significantly correlate with (p=0.219), while tinnitus was significantly correlated with hearing recovery (p=0.005), low initial threshold of hearing (0.002), down sloping graph (0.029) were positively associated with recovery.^17^

According to Atay G et al. early initiation of treatment within five days (p<.05) and flat audiogram in mid frequencies (p<0.05) show better prognosis 10 and vestibular system disorder is associated with worse outcome.^18^ In SSNHL where cause is not found, therapy including steroids and antivirals have been found of benefit.^1^,^19^ Novel approaches like neurorehabilitation using constraint-induced sound therapy has been recommended being safe and effective.^20^

## CONCLUSIONS

We conclude that the prevalence of SSNHL is 3.7% being significantly more prevalent in males and those aged 15-25 years. It is mostly characterized by severe to profound degree of hearing loss with downward sloping audiogram with no associated vertigo, tinnitus and side predilection.

### Authors’ Contribution:

**EN:** Data Analysis & Interpretation, Literature Review & responsible for integrity of the research

**GS:** Conceptualization of work, designing of research & Manuscript Writing.

**NM:** Critical revision of article.

**MNB:** Literature search and Review.
